# Raman Analyses of Laser Irradiation-Induced Microstructural Variations in Synthetic Hydroxyapatite and Human Teeth

**DOI:** 10.3390/jfb13040200

**Published:** 2022-10-25

**Authors:** Hayata Imamura, Wenliang Zhu, Tetsuya Adachi, Noriko Hiraishi, Elia Marin, Nao Miyamoto, Toshiro Yamamoto, Narisato Kanamura, Giuseppe Pezzotti

**Affiliations:** 1 Ceramic Physics Laboratory, Kyoto Institute of Technology, Sakyo-ku, Matsugasaki, Kyoto 606-8126, Japan; 2 Department of Dental Medicine, Graduate School of Medical Science, Kyoto Prefectural University of Medicine, Kamigyo-ku, Kyoto 602-8566, Japan; 3 Department of Dentistry, Kyoto Prefectural Rehabilitation Hospital for Mentally and Physically Disabled, Naka Ashihara, Johyo, Kyoto 610-0113, Japan; 4 Department of Cariology and Operative Dentistry, Graduate School of Medical and Dental Sciences, Tokyo Medical and Dental University, Bunkyo-ku, Tokyo 113-8549, Japan

**Keywords:** Raman spectroscopy, laser whitening, microstructure, synthetic hydroxyapatite, human tooth

## Abstract

The microstructural and molecular-scale variations induced by laser irradiation treatment on human teeth enamel in comparison with synthetic hydroxyapatite (HAp) were examined through Raman microprobe spectroscopy as a function of irradiation power. The results demonstrated that laser irradiation could modify stoichiometry, microstructure, and the population of crystallographic defects, as well as the hardness of the materials. These modifications showed strong dependences on both laser power and initial nonstoichiometric structure (defective content of HPO_4_), because of the occurrence of distinct reactions and structural reconstruction. The reported observations can redirect future trends in tooth whitening by laser treatment and the production of HAp coatings because of the important role of stoichiometric defects.

## 1. Introduction

Tooth treatment by laser irradiation is applied nowadays as a common and efficient approach for tooth whitening in modern dentistry [1]. Professional whitening treatment generally uses a high concentration of hydrogen peroxide for tooth bleaching, while subsequent laser irradiation increases the efficacy of the whitening treatment [2]. Tooth bleaching using peroxide-based formulations could result in a significant decrease in hardness and modulus of elasticity in the tooth enamel, as well as a change in surface micromorphology; this is due to a mineral loss of calcium content in the main component of tooth enamel, hydroxyapatite (HAp) (i.e., nonstoichiometric structure) [3,4,5]. The bleaching agents might induce loss of mechanical properties of enamel, a process partly compensated by additional laser irradiation for enamel reinforcement to improve microhardness; although laser treatment can also cause a change in the morphology and/or the crystalline structure of enamels [6]. To date, how laser irradiation affects enamel crystalline structures, in particular the stoichiometric alteration upon irradiation, as well as the effect of laser irradiation on different kinds of Hap, including natural and synthetic materials, are still insufficiently investigated.

In recent years, synthetic hydroxyapatite materials have been proposed for the reconstruction of damaged tooth and bone zones because of their excellent biocompatibility and bioactivity [7,8,9,10]. The biocompatible white layer applied as a thin surface cover on tooth enamel could adhere to the tooth surface without affecting or chemically altering the deeper tooth tissue, but assumed to reflect more light than the more transparent natural enamel to achieve the whitening effect [11]. Hydroxyapatite coatings can be achieved by laser irradiation, suggesting that this method may be used for repairing the damaged enamel surface after power bleaching [12,13]. Despite that, it has been known that both the organic component and the inorganic minerals in tooth enamel can be affected by the marked rise of local temperature induced by laser irradiation [14].

In a previous study, we have shown that thermal treatments in air hydroxyapatite ceramics revealed a significant temperature dependence of the microstructure. This is because hydroxylation and different kinds of dehydroxylation reactions may compete at different temperatures in nonstoichiometric materials [15]. As a result, the structural reconstruction results in a gradual increase of material hardness with annealing temperature. The obtained results suggested that practical whitening treatments by laser irradiation for human teeth, which generally causes a local heating effect on the tooth during laser irradiation, might also involve a structural variation of HAp in the tooth after whitening treatments. To quantitatively analyze the effects of laser irradiation on HAp structures, in this study, laser irradiation experiments were carried out on two types of hydroxyapatite ceramics and two human teeth at different powers, and the structural variations of HAp in these samples after laser irradiation were investigated by Raman microprobe spectroscopy. Raman spectroscopy is effective in evaluating and mapping material microstructural and stoichiometric alterations according to the change in the spectral morphology [16,17,18], and in this study, the spectral bandwidth of the crystalline phase and the relative intensities associated with defect and amorphous structures of HAp were observed to exhibit clear variations at the irradiated locations as a function of the laser power. The results revealed that the HAp microstructure showed a significant dependence on laser power as well as on the initial degree of the stoichiometry of the materials.

## 2. Experimental Procedures

In this study, two HAp ceramics and two human teeth (sound-erupted molar teeth) were used for laser irradiation. The HAp ceramics had a nonstoichiometric structure Ca_10−*x*_(HPO_4_)*_x_*(PO_4_)_6−*x*_ (OH)_2−*x*_ [15], and were different in initial composition (i.e., the value of the content of HPO_4_, *x*, denoted as TYPE I and II). The tooth samples were cut into two pieces and laser irradiation was performed on the enamel region of the cross-section. All subjects gave their informed consent for inclusion before they participated in the study. The study was conducted per the Declaration of Helsinki, and the protocol was approved by the Ethics Committee of the Department of Dentistry at University Hospital, Kyoto Prefectural University of Medicine or Kyoto Prefectural Rehabilitation Hospital For Mentally and Physically Disabled (project identification code: ERB-C-136, RBMR17 and RBMR19).

**Figure 1:**
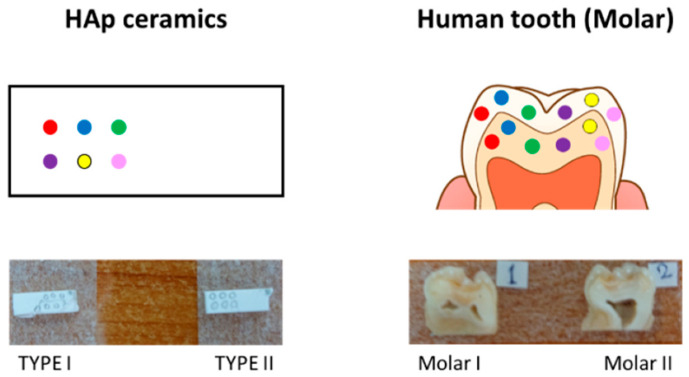
Schematic of laser irradiation on the two kinds of samples (the spots represent the irradiated locations using different laser powers).

An Er: YAG laser (Erwin AdvErl Evo, J. Morita, Kyoto, Japan) with a wavelength of 2940 nm was used for the irradiation. Single pulses of different powers (30, 50, 80, 100, 150, and 300 mJ) were manually irradiated at different locations in a light contact form using a 0.6 mm diameter curved flat contact probe tip (C600F, Morita Corporation, Kyoto, Japan). Accordingly, the laser spot size was ~600 μm and the energy density for the irradiation varied from ~2.6 to ~26 J/cm^2^. Similar laser irradiation experiments were also performed on the ceramic samples. Figure 1 shows a schematic draft of the laser irradiation on these two kinds of samples.

The sample morphologies were investigated by a back-scattered confocal laser microscope (Keyence, VK-X210, Osaka, Japan) using the excitation source of a 408 nm violet semiconductor laser and a 50× objective lens (numerical aperture of 0.55).

Raman spectra from the irradiated regions before and after laser irradiation were collected at room temperature using a Raman microprobe spectroscope, which is capable of both observation of surface morphology at a microscale resolution and collection of Raman spectra at selected irradiated locations. A single monochromator (T-64000, Jobin-Ivon/Horiba Group, Kyoto, Japan) equipped with a 1024 × 256 pixels CCD camera (CCD-3500V, Horiba Ltd., Kyoto, Japan) and a 532-nm Nd:YVO_4_ diode-pumped solid-state laser (SOC JUNO, Showa Optronics Co. Ltd., Tokyo, Japan) were used for the Raman measurements. The spectral resolution was around 0.7 cm^−1^ for the collected spectra. For all samples, average spectra were obtained from respective Raman mappings collected in a region of 50 × 50 μm^2^ with a step of 10 μm (i.e., 36 spectra) on the sample surfaces.

Raman imaging of the irradiated areas for the samples was carried out in a dedicated Raman device (RAMANtouch, Nanophoton Co., Minoo, Osaka, Japan), using a solid laser with a wavelength of 532 nm. The spatial resolution was 100 nm, but the spectral resolution was around 4 cm^−1^. Commercially available software (Raman Viewer, Nanophoton Co., Minoo, Osaka, Japan) was then used to generate Raman hyperspectral images from the raw spectra.

The Vickers hardness (HV) of the samples subjected to laser irradiation was measured by using a conventional Vickers indentation tester (AVK-C1, Akashi Co., Tokyo, Japan). A pyramidal diamond indenter was used to generate the indentation prints, by applying a load of 1.0 kgf for 20 s.

## 3. Results

**Figure 2:**
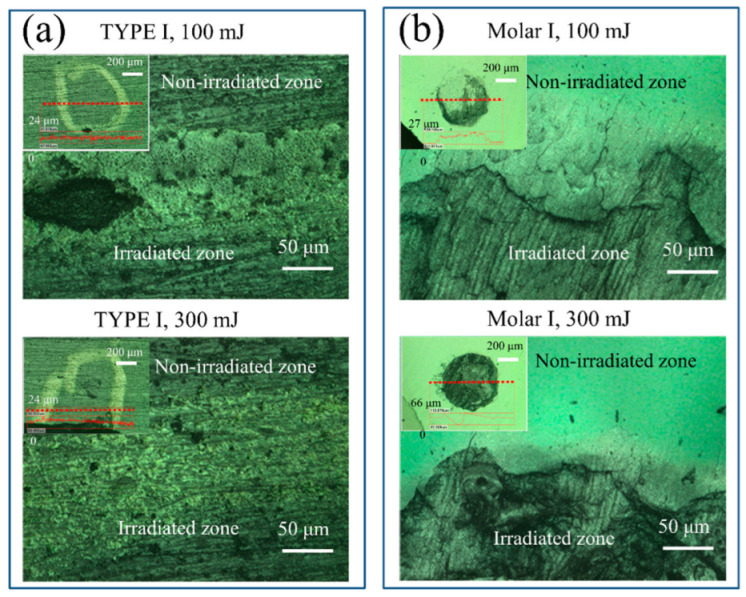
Laser microscopic images of the irradiated surfaces of (**a**) HAp ceramic and (**b**) human tooth after laser irradiation with powers of 100 and 300 mJ. The insets show respective irradiated areas using a 10× lens (irradiated zones were marked within white circles for HAp ceramic), and the profilometric changes along selected lines.

As a first step, laser microscope observations were performed to investigate the change in surface morphology of the HAp ceramic and tooth samples upon laser irradiation. The representative images of the two types of samples around the irradiated regions using powers of 100 and 300 mJ are shown in Figure 2. After laser treatment, no significant variation of surface morphology was observed for the two kinds of HAp ceramics (irradiated zones were marked within the white circles), while the tooth samples exhibited severe damage induced by the irradiation on the surfaces, especially for cases of high laser powers. In general, an increase in surface roughness could be found after laser irradiation for all samples.

**Figure 3:**
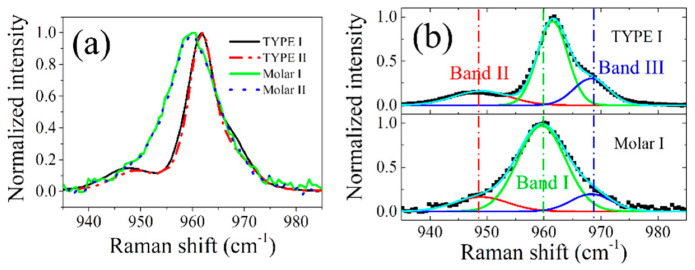
(**a**) Average Raman spectra of nonstoichiometric HAp ceramics and human teeth and (**b**) typical spectral deconvolution for these two kinds of samples.

Figure 3a compares the normalized Raman spectra collected on the untreated HAp ceramics and tooth samples in the spectral range from 935 to 985 cm^−1^. All observed bands arose from the P-O stretching *v*_1_ mode in PO_4_ tetrahedra of HAp, and spectral deconvolution of the representative spectra (Figure 3b) revealed the presence of three sub-bands, located at around 947, 961, and 969 cm^−1^. These bands correspond to the amorphous phase (band II), crystalline structure (band I), and HPO_4_ group defect (band III) in HAp, respectively [19,20,21]. Note that the two kinds of ceramics (TYPE I and II) have slightly different intensities (sub-band area) for bands II & III in the normalized spectra (higher values for TYPE I), but each sample exhibited a negligible change in the Raman spectral morphology at different locations within the sample (cf. Figure S2 in the supplementary material of Ref. [15]), indicating a compositional homogeneity throughout the sample.

In general, concerning the tooth samples, the HAp ceramics showed a higher peak position of band I (~961.5 vs. 959.5 cm^−1^), which was closer to that of single hydroxyapatite crystals (~962 cm^−1^ [19]), and a much smaller full width at half maximum (FWHM) (~5.1 vs. 8.6 cm^−1^), indicating a much higher crystallinity of HAp in the ceramic samples, since increasing FWHM denotes a decrease in crystallinity [22,23]. Moreover, compared with tooth samples, the HAp ceramics also exhibited higher values of the intensity ratio of *I*_969_/*I*_961_, especially TYPE I, suggesting a higher content of HPO_4_ defect in the non-stoichiometric structure.

**Figure 4:**
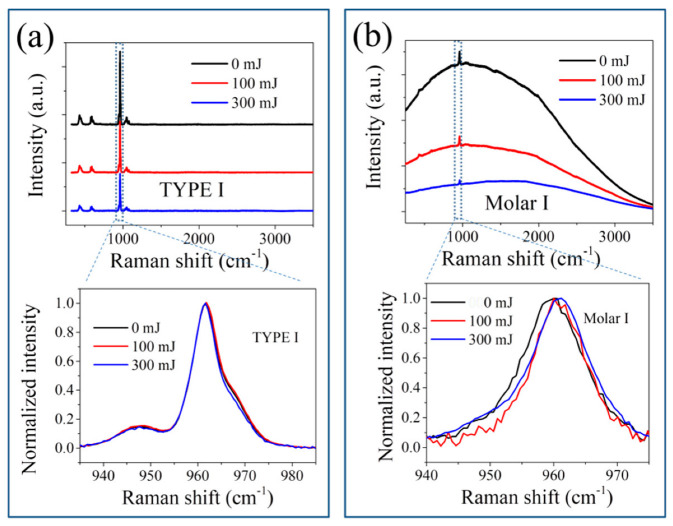
Variations of Raman spectra for (**a**) HAp ceramics and (**b**) tooth samples subjected to laser irradiation at 0, 100, and 300 mJ, with an enlargement in the range from 940 to 985 cm^−1^ after baseline subtraction.

Upon laser irradiation treatments, the HAp ceramics and tooth samples were found to exhibit distinct changes in the Raman line shape with increasing laser power. Figure 4 shows the variations of Raman spectra of the samples in the full spectral range from 350 to 3500 cm^−1^ before and after laser irradiation with powers of 100 and 300 mJ, respectively, and an enlarged plot of the *v*_1_ band after baseline subtraction. In general, the HAp ceramics showed quite a small change in the spectral line shape after laser irradiation, while the tooth samples revealed an efficient reduction in the fluorescence background induced by the presence of organic components or contamination [24,25], and a visible decrease in the intensity of the *v*_1_ band, as well as slight changes in its band position and FWHM.

**Figure 5:**
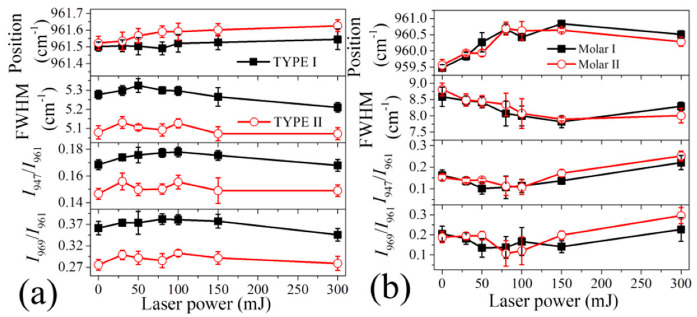
Variations of peak position and FWHM of band I, and intensity ratios of *I*_947_/*I*_961_ and *I*_969_/*I*_961_ with laser power for (**a**) HAp ceramics, and (**b**) tooth samples subjected to laser irradiation.

After baseline subtraction and spectral deconvolution, detailed changes in peak position, FWHM, and intensity ratios of *I*_947_/*I*_961_ and *I*_969_/*I*_961_ for the samples in response to the increase of laser power are shown in Figure 5. As can be seen in Figure 5a, for HAp ceramics, increasing laser power resulted in the position of band I slightly but gradually shifting to higher wavenumbers at different rates of increase, accompanied by first a minor increase and then a decrease in FWHM. Concurrently, the intensity ratios of *I*_947_/*I*_961_ and *I*_969_/*I*_961_ first increased as the laser reached 100 mJ, then decreased at higher laser powers.

In contrast, the tooth samples exhibited a peak shift of band I to higher wavenumbers as the laser power increased, but then a decrease at higher laser powers, while FWHM showed a trend of initially decreasing and then increasing at higher powers (Figure 5b). The two intensity ratios also showed a similar trend of variation to that of FWHM, i.e., an initial decrease as laser power reached 100 mJ, and then an increase at higher powers.

Because HAp ceramics have a non-stoichiometric structure, such observed differences are considered to be due to their different initial compositions before laser irradiation, as discussed later.

**Figure 6:**
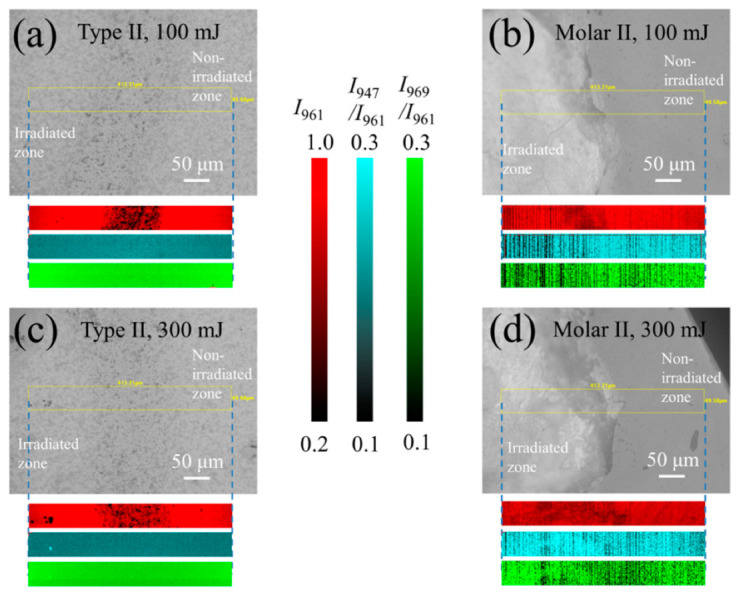
Surface images of (**a**,**c**) the HAp ceramic and (**b**,**d**) the tooth sample subjected to laser irradiation using powers of (**a**,**b**) 100 and (**c**,**d**) 300 mJ, and respective Raman maps of *I*_961_ and intensity ratios of *I*_947_/*I*_961_ and *I*_969_/*I*_961_.

To further investigate the defect population and structural alteration upon laser irradiation, Raman microscopic imaging was carried out on these samples in a selected region, including both the irradiated zone and the non-irradiated zone. Figure 6 shows the surface images of the HAp ceramic and the tooth sample after laser irradiation using powers of 100 and 300 mJ, as observed under a Raman microscope, and the respective Raman maps of the 961 cm^−1^ band intensity, *I*_961_, as well as intensity ratios of *I*_947_/*I*_961_ and *I*_969_/*I*_961_.

As can be seen in Figure 6, for the HAp ceramic, despite an observed decrease of *I*_961_ at the boundary due to the presence of the introduced mark (also cf. inset to Figure 2a), the two intensity ratios showed an indiscernible variation within the irradiated zone. This is because the lower spectral resolution of this machine for imaging is incapable of revealing the slight variations of these values shown in Figure 5a. In contrast, for the tooth samples, the irradiated zones could be clearly observed from the microscope and showed distinct variations from the non-irradiated zone. The intensity ratios of *I*_947_/*I*_961_ and *I*_969_/*I*_961_ showed a decrease (i.e., darker in contrast) in the zone irradiated with a laser power of 100 mJ, while these values increased at 300 mJ. Despite a lower spectral resolution, the observations made by Raman imaging are consistent with the results given in Figure 5b.

**Figure 7:**
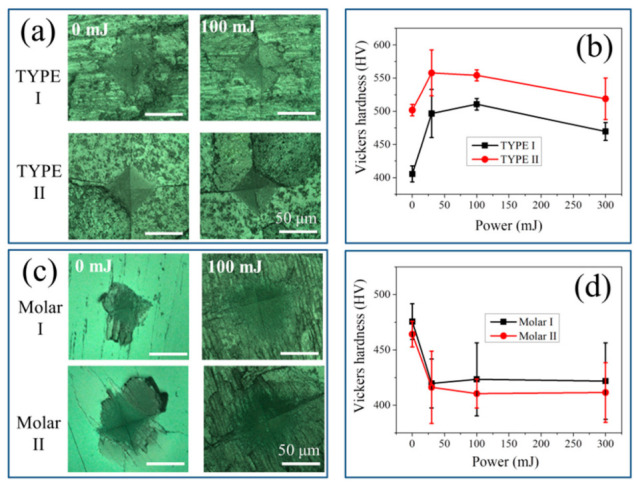
Laser microscopic images of the indentation prints on the surfaces of (**a**) HAp ceramics and (**c**) tooth samples with and without laser irradiation using a power of 100 mJ, and plots of Vickers hardness as a function of laser power for (**b**) HAp ceramics and (**d**) tooth samples.

The Vickers indentation method was then applied to investigate the influence of laser irradiation on material hardness. Figure 7a,c show representative micrographs of the prints generated on the two types of samples with and without laser irradiation using a power of 100 mJ. All indentation prints exhibited similar square-like profiles for the investigated samples. Unlike the ceramic sample, the tooth samples did not exhibit any crack generation around the prints. As shown in Figure 7b,d, according to the variation of the mean diagonal length of the print in response to the laser power, the values of the Vickers hardness and HV could be calculated at different laser powers for HAp ceramics and tooth samples, respectively.

The curves representing Vickers hardness values revealed distinct trends: for both types of HAp ceramics, the Vickers hardness first increased when the samples were irradiated with low powers, but became saturated or even slightly decreased at high powers. In general, the TYPE II samples exhibited higher HV values than TYPE I. On the other hand, the tooth samples showed a decrease in HV with increasing laser power, and no significant difference in the HV variation curve could be found between molar I and molar II.

## 4. Discussion

Because of the incorporation of defects in the lattice (e.g., substitutional ions & vacancies), the Ca/P molar ratio of both natural and synthetic HAp materials usually deviated from the stoichiometric value, 1.67 of Ca_10_(PO_4_)_6_(OH)_2_ [26,27]. In most cases, the main contributors for the off-stoichiometry are proton incorporation into the HAp lattice as interstitial proton (H_i_^•^) and substitutional proton [28], and the presence of energetically favorable vacancies, e.g., OH, and Ca vacancies in the lattice [27,29,30]. The compound that comprises both PO_4_ and HPO_4_ groups, vacancies of calcium and hydroxyl, and H_2_O moieties, is generally denoted by a chemical formula of Ca_10−*x*_(HPO_4_)*_x_*(PO_4_)_6−*x*_(OH)_2−*x*_(H_2_O)*_x_* [31,32].

In our previous paper, we have shown that the microstructure and chemical composition of nonstoichiometric hydroxyapatite ceramics showed a strong temperature, due to the existence of competition between hydroxylation and dehydroxylation reactions upon thermal treatment [15].

At low temperatures, along with hydroxylation reactions to incorporate H_2_O into HAp, migration of hydrogen and hydroxyl ions can occur to respectively fill in the vacancies of calcium and hydroxyl (V_Ca_ and V_OH_), which results in a structural reconstruction and the formation of Ca-O-H and P-O-H, while the OH fraction from H_2_O moieties is reduced.

(1) $$\begin{array}{c} {\mathrm{Ca}}_{10-x}{\left({\mathrm{PO}}_{4}\right)}_{6-x}{\left({\mathrm{HPO}}_{4}\right)}_{x}{\left(\mathrm{OH}\right)}_{2-x}{\left(H_{2}O\right)}_{z}+yH_{2}O\;\to {\;\mathrm{Ca}}_{10-x}{\left({\mathrm{PO}}_{4}\right)}_{6-x-z-y}{\left({\mathrm{HPO}}_{4}\right)}_{x+z+y}{\left(\mathrm{OH}\right)}_{2-x+z+y}\\ \;\overset{x=y+z}{\to }\;{\mathrm{Ca}}_{10-x}{\left({\mathrm{PO}}_{4}\right)}_{6-2x}{\left({\mathrm{HPO}}_{4}\right)}_{2x}{\left(\mathrm{OH}\right)}_{2} \end{array}$$

Consequently, by increasing the laser power from 0 to 100 mJ (i.e., increase of local surface temperature) for the HAp ceramics, the intensities of the Raman bands related to amorphous or disordered structures and HPO_4_ defect (i.e., bands II and III) increases with respect to band I, and the peak of the crystalline phase shows an increase in FWHM because of the induced lattice disorder (cf. Figure 5a), as the *c* axis OH groups may undergo disorder in the column [33].

However, at higher laser powers, or higher temperatures, dehydroxylation reactions may prevail by the removal of hydroxyl groups from water moieties and also from neighboring Ca-O-H and P-O-H to form P-O-Ca, and then a lattice structural reconstruction occurs to improve the crystallinity of the ceramics.

(2) $$\begin{array}{c} {\mathrm{Ca}}_{10-x}{\left({\mathrm{PO}}_{4}\right)}_{6-2x}{\left({\mathrm{HPO}}_{4}\right)}_{2x}{\left(\mathrm{OH}\right)}_{2}\to {\;\mathrm{Ca}}_{10-x}{\left({\mathrm{PO}}_{4}\right)}_{6-2x+2y}{\left({\mathrm{HPO}}_{4}\right)}_{2x-2y}{\left(\mathrm{OH}\right)}_{2-2y}+2yH_{2}O\\ \;\overset{\;x=y}{\to }\;{\mathrm{Ca}}_{10-x}{\left({\mathrm{PO}}_{4}\right)}_{6}{\left(\mathrm{OH}\right)}_{2-2x}+2xH_{2}O \end{array}$$

Accordingly, the distorted PO_4_ tetrahedral structure can be recovered in connection with Ca at the columnar site because of the reduction of proton-related defects, and a relatively stable defect complex will be formed by combining the negatively charged Ca^2+^ vacancy with the positively charged OH^−^ vacancy, (V_Ca_, V_OH_). Therefore, a decrease of the two intensity ratios and the peak width can be expected with increasing the laser power, as shown in Figure 5a.

Note that for untreated HAp ceramics, the competition of the above reactions (1) and (2) should strongly depend not only on temperature but also on the initial content of HPO_4_ (or hydroxyl vacancy). As shown in Figure 3a, the TYPE I HAp ceramic sample exhibited higher values of *I*_969_/*I*_961_ and *I*_947_/*I*_961_ than TYPE II, indicating a higher content of HPO_4_ defect and a higher lattice disorder. Therefore, because of the higher fraction of amorphous structure and defects, the untreated TYPE I sample should possess a lower Vickers hardness than TYPE II, as confirmed in Figure 7b. Upon laser irradiation with low powers, as expressed by Equation (1), the reaction kinetic was relatively insignificantly influenced by the amount of defect, *x*, but the total content of incorporated hydroxyl and HPO_4_ in the lattice was determined by *x*. Therefore, the TYPE I sample was considered to show a larger variation of the two intensity ratios relative to TYPE II upon laser irradiation, as demonstrated in Figure 5a, although the observed difference was not so pronounced because of data scattering. Note that it is clearer to find such an expected difference from the change of Vickers hardness, as TYPE I showed a larger difference between the maxima and the minima, than Type II (Figure 7b).

The alteration of the mechanical hardness of the materials by the laser power is mainly owing to a structural reconstruction induced by the above reactions: <i> at low temperatures the hydroxyl ions migrate to the *c*-axis hydroxyl vacancy, and <ii> at higher temperatures, the hydrogen terminal of P-O-H is removed for a higher-degree lattice interconnection, and the water loss from the lattice causes an increased density [15,34].

In the cases of tooth samples, a much larger *v*_1_ bandwidth of the calcium phosphate crystalline phase (crystallites of minerals, 95–96% of enamel) than that of the ceramic ones could be seen (Figure 3b). This is related to a lower crystallinity and the substituents in the lattice of enamel by impurities, including Na, K, Mg, Sr, CO_3_, and F. Despite constituting only a small fraction, the impurities in the enamel are particularly effective at modifying or regulating the response of the minerals, especially the strength needed to protect the teeth from acidic environmental agents compared with that of pure hydroxyapatite [35,36]. As shown in Figure 3, the tooth samples show a lower value of *I*_969_/*I*_961_, indicating a smaller fraction of hydrogen defect POH and hydroxyl vacancy, *x*. Note that the incorporation of F^−^, and CO_3_^2^^−^ ions at hydroxyl sites can also cause a reduction of hydroxyl vacancy. Accordingly, because of the smaller defect content, *x* for hydrogen or hydroxyl incorporation in the lattice, and the high amount of water in enamel (~3–4%), a minor fraction of the incorporation of H_2_O in HAp by hydroxylation reactions can be expected. Concerning dehydroxylation, these reactions are dominant during laser irradiation:

(3) $$\begin{array}{c} \;{\mathrm{Ca}}_{10-x}{\left({\mathrm{PO}}_{4}\right)}_{6-x}{\left({\mathrm{HPO}}_{4}\right)}_{x}{\left(\mathrm{OH}\right)}_{2-x}{\left(H_{2}O\right)}_{z}\;\overset{\Delta }{\to }{\;\mathrm{Ca}}_{10-x}{\left({\mathrm{PO}}_{4}\right)}_{6-x+y}{\left({\mathrm{HPO}}_{4}\right)}_{x-y}{\left(\mathrm{OH}\right)}_{2-x-y}+\left(y+z\right)H_{2}O\;\\ \;\overset{x=y}{\to }\;{\mathrm{Ca}}_{10-x}{\left({\mathrm{PO}}_{4}\right)}_{6}{\left(\mathrm{OH}\right)}_{2-2x}+\left(x+z\right)H_{2}O \end{array}$$

Therefore, accompanying the reduction in the hydrogen defect of P-O-H and the following structural reconstruction, as well as a possible loss of the CO_3_^2^^−^ components at low temperatures [37], the crystallinity of the enamel is increased at low laser powers, reflected by a decrease of two intensity ratios, *I*_969_/*I*_961_ and *I*_947_/*I*_961_ and the peak width with increasing laser power (cf. Figure 5b and Figure 6b).

Nevertheless, dehydroxylation to remove two *c*-axis OH ions may occur at relatively high laser powers or temperatures because of the low formation energy of the vacancy defect complex, (V_H_+,V_OH_^−^) [38,39].

Ca_10−*x*_(PO_4_)_6_(OH)_2−2*x*_→ Ca_10−*x*_(PO_4_)_6_ (OH)_2−2*x*−2*y*_O*_y_*+ *y*H_2_O (4)

Further removal of hydroxyl groups along the *c* axis with spontaneous formation of vacancy pairs can result in a lattice disorder, and increased protonic conductivity [40]. Note that due to electrostatic interactions, the cations (Ca^2+^ atoms) surrounding the positive effective charge vacancy V_OH_^−^ will show an outward relaxation to distort the lattice. Therefore, both the severe lattice distortion and the structural disorder can cause an increase of the intensities of bands II and III relative to band I (cf. Figure 5b and Figure 6d), and a broadening of band I (cf. Figure 5b) at high laser powers. In the case of laser irradiation with high-energy density on human teeth, apatite modifications, including phase transformation to other calcium phosphates, together with the reductions in contents of water, protein, and carbonate, may occur owing to the induced high temperatures. These factors can also cause an alteration of the material hardness and severe damage to the tooth [41,42,43].

It should also be noted that since the tooth enamel contains ~1% organic material, mainly two unique classes of proteins: amelogenins and enamelins [44]. Upon laser irradiation, the decomposition and oxidation of the proteins could cause damage to the tooth surface because the mechanical properties of tooth enamel are significantly regulated by the structural and compositional characteristics of the minor protein component [45]. Consequently, with the removal of water and decomposition of proteins, the alteration and shrinkage of the crystalline lattice and the surface reconstruction caused a formation of microcracks and an increased roughness in the irradiated regions, as observed in Figure 2b and Figure 7c. The presence of microcracks and surface structural relaxation could thus result in a slight decrease of the Vickers hardness of the tooth samples, even though an increase in the crystallinity was observed (Figure 7d).

Finally, in this study, laser irradiation was performed on the tooth cross sections instead of the enamel surface to reduce the influence of surface morphology on <i> the laser intensity distribution within the large spot (0.6 mm) during irradiation, <ii> the Raman intensity variation owing to laser defocusing during Raman mapping, and <iii> the alignment of generated indentation prints. Moreover, the use of a flat surface can make all the above analyses easier. However, it should be noted that the mechanical properties of enamel with a rod and inter-rod structure have been reported to depend on the location within the microstructure, showing high hardness and elastic modulus parallel to the rod axis [46]. In this study, the regions for laser irradiation and Vickers indentation were all on the cross sections of the tooth enamels (Figure 1), and thus the anisotropy of mechanical hardness caused by microstructure in the enamel was neglected.

In summary, this paper demonstrated that Er:YAG laser irradiation treatment of HAp-based materials can modify the chemical composition, microstructure and defects, as well as mechanical hardness of the materials, showing a dependence not only on laser power, but also on initial composition (i.e., the content of HPO_4_, *x*). These observations could help in designing future tooth whitening by laser treatment and the production of HAp coating, because of the important role of chemical nonstoichiometry in HAp materials [28,47]. For laser whitening treatments, since the whitening agents, bleaching gel, and laser irradiation can cause an alteration to the microstructure and mechanical properties of HAp or enamel, the distinct variations of the Vickers hardness in HAp ceramics and molar teeth subjected to laser irradiation for enamel reinforcement suggest a need for careful consideration of both the stoichiometric alteration of the teeth by the whitening treatment and the power of clinic laser irradiation. For the preparation by laser irradiation of synthetic hydroxyapatite materials proposed for enamel coating to have an intrinsic “whitening effect” and reconstruction of the damaged tooth and bone zones, the microstructural and stoichiometric alterations, as well as the properties can be controlled by the power of irradiation.

## 5. Conclusions

In this paper, Raman microprobe spectroscopy was applied to evaluate the variations in microstructures subjected to irradiation treatments using an Er:YAG laser with different power settings in HAp ceramics and human teeth. Unlike the ceramic samples, laser irradiation showed marked variations of the Raman spectra of human teeth with laser power by reducing the background fluorescence owing to the presence of organic materials and contaminants, indicating an effective surface treatment. However, it also revealed clear variations of the spectral bandwidth of the crystalline phase and the relative intensities associated with defect and amorphous structures of HAp at the irradiated locations as a function of the laser power. Raman microscopic imaging performed on these samples in a selected region, including both irradiated and non-irradiated zones, confirmed the observed variations in the irradiated zones on the tooth samples. The nonstoichiometric structure, including HPO_4_ in the lattice in HAp ceramics and teeth, caused distinct dependences of the chemical composition, crystallinity, and material hardness on laser power, as well as on its initial defect content of HPO_4_, *x* This is because of the occurrence of different reactions and structural reconstruction at different powers of laser irradiation. Despite the increase of HAp crystallinity with increasing laser power for human teeth, the formation of microcracks and increased roughness because of irradiation damages resulted in a decrease in the hardness of the tooth, unlike ceramics. Accordingly, because of the important role of defects, the obtained results can direct future tooth whitening by laser treatment and the production of HAp coatings for dental applications.

## Data Availability

No new data were created or analyzed in this study. Data sharing is not applicable to this article.
